# Supplementary material for: Antimicrobial Activity of Silver-Treated Bacteria against Other Multi-Drug Resistant Pathogens in Their Environment
Source: Antibiotics (Basel). 2020 Apr 15;9(4):181. doi: 10.3390/antibiotics9040181 (PMC7235873; doi:10.3390/antibiotics9040181)
Supplement: Supplementary file 1 [file antibiotics-09-00181-s001.pdf]

Supplementary data:

Table S1. Antimicrobial activity of Silver treated bacteria (killed bacteria and the supernatant) on *Ps. aeruginosa, E. coli* O157:H7 and MRSA using well agar diffusion method.

	**Inhibition zones** ± S.D (mm)		**P value**
	**Silver killed bacteria**	**Supernatant**	
Ps. aeruginosa	**24± 0.14**	**23± 0.31**	**> 0.05**
**MRSA**	**18 ± 0.24**	**20 ± 0.16**	**> 0.05**
E. coli O157:H7	**24± 0.33**	**25 ± 0.25**	**> 0.05**

***P* values are significant at < 0.05**
